# Establishment and development of the Center of Plant Systems Biology and Biotechnology in Plovdiv, Bulgaria

**DOI:** 10.12688/openreseurope.16514.1

**Published:** 2023-09-07

**Authors:** Tsanko Gechev, Petar Kazakov, Asia Ivanova, Tsvetomira Ivanova, Marina Mircheva, Vasil Kolev, Daniela Ganeva, Veneta Tabakova-Komsalova, Margarita Ruseva, Elitsa Kantardjieva, Vesela S. Kazashka

**Affiliations:** 1 Center of Plant Systems Biology and Biotechnology, Plovdiv, Plovdiv Province, Bulgaria; 2 University of Plovdiv, Plovdiv, 4000, Bulgaria; 3 Academy of Music, Dance, and Fine Arts, Plovdiv, 4000, Bulgaria; 4 Maritsa Vegetable Crops Research Institute, Plovdiv, 4004, Bulgaria

**Keywords:** Biotechnology, Centers of Excellence, Plant Systems Biology, Vegetable Breeding

## Abstract

The Bulgarian research landscape, presented mainly by the research institutes that are part of the Bulgarian Academy of Sciences and the Agricultural Academy, needs diversification to match the research and innovation potential of the other European Union (EU) countries. This article describes the establishment of the Center of Plant Systems Biology and Biotechnology (CPSBB), a new innovative type of independent research organization that is changing the research landscape in Bulgaria. Supported by the EU Commission, Bulgarian Government, and Plovdiv Municipality, CPSBB has quickly become the leading plant science institute in Bulgaria, creating knowledge in diverse fields such as bioinformatics, biotechnology, genetics and genomics, metabolomics, and systems biology. We outline the organizational structure of CPSBB, the development of its infrastructure, and its scientific productivity. Finally, we compare CPSBB with other similar research establishments in Europe and we conclude that such new types of institutes have a bright future in Bulgaria due to their operational flexibility, productivity, and connections with academia and industry.

## Introduction

In 2013, the Horizon 2020 framework programme of the European Commission launched a new Teaming instrument, intended to improve the research performance and increase investment in EU member and associated countries with lower research and innovation index ( H2020-WIDESPREAD-2014-2015). The enhancement of the research and innovation potential in the so called Widening countries would be implemented by creating new or upgrading existing centers of excellence in the Widening countries and teaming up with leading European institutes.

Bulgaria is one of the Widening countries, together with Portugal, Luxemburg, the countries that joined the European Union (EU) after 2004, and several Horizon 2020 associated states. A thorough analysis of the Bulgarian research ecosystem, presented in the Bulgarian National Strategy for Development of Scientific Research, identified that most of the Bulgarian research institutes, which are part of either the Bulgarian Academy of Sciences or the Agricultural Academy, suffer from insufficient funding, infrastructure that needed upgrading, low personnel salaries, and little connections with the industry, ultimately resulting in massive brain drain and low international competitiveness. Furthermore, as outlined in the Bulgarian Innovation Strategy for Smart Specialisation 2014-2020, there was a need for a new research institute to study the rapidly evolving field of plant systems biology and to apply plant biotechnology approaches to solve important challenges such as food production or production of plant-derived metabolites with medical and pharmaceutical applications.

In order to address these issues, a team of Bulgarian and German scientists established the Center of Plant Systems Biology and Biotechnology (CPSBB) in 2015 as a new fully independent research organization with the aim to perform fundamental and applied research up to the highest international standards, to connect academia with industry in Plovdiv, and to position Bulgaria at the forefront of European plant science ( Gechev *et al.*, 2020). The catalyst for this development was the Horizon 2020 project PlantaSYST, together with follow-up co-funding from the Bulgarian Operational Programme “Science and Education for Smart Growth” (OP SESG) and support from Plovdiv Municipality. CPSBB is innovative for Bulgaria in many ways, as it is fully independent from the two large Academies, international, and very flexible operationally. The knowledge it generates equals the best science centers in Europe. CPSBB was created using the example of the Teaming partner Max Planck Institute of Molecular Plant Physiology, Potsdam-Golm, Germany, while aligning the administrative and technical structures to the local environment and the Bulgarian Law. Here we describe the multi-step creation of CPSBB and its establishment as a new player in the Bulgarian research landscape.

## Establishment of CPSBB

**Figure 1.**
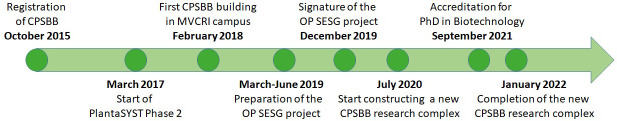
Establishment and key development moments of the Center of Plant Systems Biology and Biotechnology (CPSBB).

The initial funding for the establishment of CPSBB came from the first phase of the Teaming project PlantaSYST (2014 – 2015), which aimed to prepare a robust 10-year business plan for the operation of CPSBB. CPSBB was registered in the Court of Plovdiv in October 2015 as a fully independent research institute ( Figure 1). This full autonomy, in the spirit of the new Teaming initiative, was actually creating a new type of research establishment that was changing the research landscape in Bulgaria. In fact, CPSBB is not under the umbrella of the Bulgarian Academy of Sciences or the Agricultural Academy, which allows fast decision making and operational flexibility. At the very beginning, the Maritsa Vegetable Crops Research Institute (MVCRI), one of the PlantaSYST partners, provided CPSBB with an office and then with one building. In parallel, the Bulgarian Government was working on the funding instrument that would provide financial resources for infrastructure development of CPSBB and created the OP SESG, which opened the co-funding scheme later on. At the same time, Plovdiv Municipality and the Mayor’s office provided much needed support in terms of providing land for the new CPSBB research complex. In 2017, in a very short period of time at the start of the second phase of PlantaSYST, the whole organizational structure of CPSBB and all of its research and service departments were established.

## Structure and management of CPSBB

### Organizational structure of CPSBB

The General Assembly (GA) is the supreme body of CPSBB which determines the general policy of CPSBB, makes strategic decisions, appoints the executive bodies (Director and the Executive Boart), and approves the Heads of Departments. The executive bodies implement the general policy and the strategic decisions of CPSBB. An International Scientific Advisory Board (ISAB) was established at the very beginning of PlantaSYST to monitor all project activities and CPSBB development. The ISAB is a fully independent external body that provides objective advice on the management, infrastructure development, and research activities of CPSBB. Further down, the CPSBB structure is organized in administrative, technical, and IT units, as well as research and service Departments. The CPSBB Administration manages the administrative and financial management of CPSBB. It also takes care of communication with national and EU authorities (National Revenue Agency, National Statistics Institute, National Evaluation and Accreditation Agency) and the doctoral program in Biotechnology. Among the huge workload is also the management of many national and international projects ( Table 1), supporting in this way the fundamental and applied research conducted by the CPSBB Departments.

**Table 1.** Current national and international projects with participation of the Center of Plant Systems Biology and Biotechnology (CPSBB).

Project title/Acronym	Funding body	Duration	Role of CPSBB
PlantaSYST	Horizon 2020	2017 – 2025	Coordinator
BG05M2OP001-1.003-0001-C01	OP SESG	2019 – 2023	Coordinator
RESIST	Horizon 2020	2019 – 2024	Coordinator
ScienceAgainstInfodemic	ACF	2021 – 2023	Beneficiary
AgroDigiRise	Horizon Europe	2022 – 2025	Beneficiary
CAFTA	NSF of Bulgaria	2022 – 2027	Coordinator
K-TRIO	Horizon Europe	2023 – 2024	Beneficiary
CropPrime	Horizon Europe	2023 – 2027	Beneficiary
NatGenCrop	Horizon Europe	2023 – 2027	Coordinator
Motivation	NSF of Bulgaria	2023 – 2025	Coordinator
AbioStressTolerance	NSF of Bulgaria	2023 – 2025	Coordonator
EpiFlowScen	NSF of Bulgaria	2023 – 2025	Coordinator
ChARomics	NSF of Bulgaria	2023 – 2025	Coordonator
PlantMetals	NSF of Bulgaria	2022 – 2024	Coordonator
BenBedPhar	NSF of Bulgaria	2023 – 2025	Coordonator
BOOSTER	Horizon Europe	2023 – 2028	Beneficiary
BELIS	Horizon Europe	2023 – 2028	Beneficiary
BG-175467353	NSF of Bulgaria	2023	Coordinator
HelthyDiets4Africa	Horizon Europe	2023 – 2029	Beneficiary

### CPSBB research and service departments

The CPSBB departments conduct fundamental and applied research, as well as provide services to partner organizations and external clients ( Gechev *et al.*, 2020). Below we briefly describe the main activities of the CPSBB departments.

The Department of Bioinformatics performs its own research related to sequencing and analysis of plant genomes. At the same time, it also provides support to the other research departments at CPSBB in all major aspects related to the analysis of “omics” data sets (for example, RNA-sequencing, whole-genome sequencing, integrating transcriptome and metabolome datasets, and others) ( Dong *et al.*, 2019; Lyall *et al.*, 2020).

The Department of Molecular Stress Physiology studies the molecular and genetic mechanisms behind plant abiotic and oxidative stress tolerance. Research directions include extremophile plants, such as the desiccation, low temperature, or UV radiation tolerant species ( Durgud *et al.*, 2018), identification of new plant genes that modulate abiotic and oxidative stress tolerance ( Sujeeth *et al.*, 2020), and utilizing an effective, environmentally friendly molecular priming technology in which treatment of plants with biostimulants induces genes and metabolites that protect from subsequent stress ( Kerchev *et al.*, 2020; Omidbakhshfard *et al.*, 2020; Rasul *et al.*, 2021; Staykov *et al.*, 2021). More recently, the Department of Molecular Stress Physiology extended its abiotic stress research to include salinity ( Ali *et al.*, 2023).

The work at the Department of Plant Development is focused on transcription factors that govern various developmental processes, as well as genes that modulate plant ageing ( Kanojia *et al.*, 2020).

The Department of Vegetable Breeding, together with the Department of Crop Quantitative Genetics and department of Plant Metabolomics, studies the molecular genetics of fruit shape, size, and color in vegetable crops, as well as genes that regulate secondary metabolism and interaction with the environment ( Nankar *et al.*, 2020a; Nankar *et al.*, 2020b; Tong *et al.*, 2022).

The work at the Plant Cell Biotechnology focuses on investigating plant molecules with potential use in medicine and pharmacy (e.g. with anti-obesity, anti-inflammatory, or immunoregulatory activities), as well as development of biotechnological tools for their sustainable mass production ( Amirova *et al.*, 2021; Mladenova *et al.*, 2022; Savova *et al.*, 2023; Vasileva *et al.*, 2021).

The Department of Technology Transfer and IP Management has important roles to connect CPSBB researchers with the industry, translating the fundamental research into practical applications, and to protect the CPSBB intellectual property rights. Multiple connections with companies from Bulgaria and abroad have been established and bi-lateral cooperations, framework partnership agreements, and contracts were signed.

The Department of Funding supports conceptualization, development, and implementation of new project proposals and grant applications. Many projects were awarded to CPSBB either by national or by international funding organizations until now ( Table 1).

## Development of CPSBB in the period 2017 – 2023

### Infrastructure development

Creating a new research institute from scratch is challenging. Both infrastructure and human capacity needs to be developed, which normally takes many years. Due to a robust development plan, CPSBB managed to set up the initial infrastructure and the core research personnel in just one year after the start of PlantaSYST Phase 2. The Maritsa Vegetable Crops Research Institute, one of the PlantaSYST partners, provided CPSBB with an old building which was renovated very fast and converted into a functional facility with offices, seminar room, laboratories, and plant growth rooms ( Figure 1). In parallel, Plovdiv Municipality provided 23,500 m ^2^ of land in the Trakia residential district of Plovdiv for construction of a brand new research complex and the Bulgarian Government provided 30,000,000 BGN (15,300,000 EUR) for constructing the new research complex of CPSBB and state of the art equipment. The construction of the new CPSBB campus began in July 2020 and was completed in record time in January 2022 ( Figure 1). The new research complex provides spacious offices, a large conference hall for 300 people, four large seminar rooms, laboratories, rooms for specialized equipment, and two large greenhouses with a total area of 4,000 m ^2^. The new research complex of CPSBB was named the “ Building of the year 2022”, a prestigious prize awarded by an independent committee of 33 architects. Now, the new research complex of CPSBB and the previous one function in parallel, providing excellent facilities to the CPSBB personnel.

Regarding the scientific equipment, initial support was provided by the Bulgarian Government through the program National Roadmap of Research Infrastructure (NRRI). Through NRRI, CPSBB acquired modern equipment for its molecular biology, biochemistry, and plant physiology laboratories. In parallel, the Max Planck Institute of Molecular Plant Physiology in Potsdam-Golm provided two donations of basic scientific equipment. The bulk of the scientific equipment, though, including the state of the art metabolomics and biotechnology machines (ICP-MS, GC-MS, UHPLC-MS, pilot-scale bioreactor, confocal laser microscope, state of art plant growth chambers, etc.) was purchased through the OP SESG co-funding project after the construction of the new CPSBB research complex. With these purchases, the new research complex became fully functional, working in parallel with the CPSBB building located in the MVCRI campus.

### Developing human potential

Attracting and keeping excellent researchers as well as professional administrative and technical staff is extremely difficult in Bulgaria; this was one of the challenges identified during the initial Strengths, Weaknesses, Opportunities, Threats (SWOT) analysis. The main problems for that are low salaries in most of the Bulgarian research institutes, the underdeveloped research infrastructure, and the low level of internationalization. Furthermore, Bulgaria has never been considered as a top research destination. CPSBB has addressed all of these challenges by offering very competitive salaries, by developing modern research infrastructure as good as any of the top level European research institutes, and by advertising Bulgaria as an attractive research destination. Advantages of Bulgaria and Plovdiv in particular, include low taxes, low living expenses, a very low crime rate in Plovdiv, a rich cultural life (Plovdiv is the 2019 European Culture Capital), very good infrastructure, and excellent living conditions for family and children. These, in combination with the competitive salaries and excellent research infrastructure, have attracted a number of foreign researchers to CPSBB. Currently, according to the percentage of foreign researchers, CPSBB is the most international Bulgarian research institute.

 In the first years since its establishment, CPSBB set up the rules for employment of administrative, technical, and research personnel. CPSBB is an equal opportunity employer and the recruitment is based on merits, regardless of nationality, gender, or religion. At the very beginning, experienced administrative and technical staff was employed in order to get CPSBB running. In parallel, recruitment processes for postdoctoral scientists and technicians were initiated and several excellent scientists were employed. As a result, all administrative and technical units, as well as the research departments, were secured with the necessary personnel. More recently, and as an evolution that coincided with the development of CPSBB infrastructure, positions for new group leaders were announced. This also included the establishment of the newest research department “Crop Quantitative Genetics”, established in 2023 with the recruitment of Dr. Saleh Alseekh as Head. The new high profile group and department leaders have contributed to the image of CPSBB as a leading research organization in Bulgaria.

In 2021, CPSBB received a positive evaluation by the National Evaluation and Accreditation Agency and was accredited to deliver PhD degrees in Biotechnology ( Figure 1). This important development allowed enrolling new PhD students, strengthening the human potential of CPSBB, and applying for research grants to the National Science Fund.

### Scientific development

**Figure 2.**
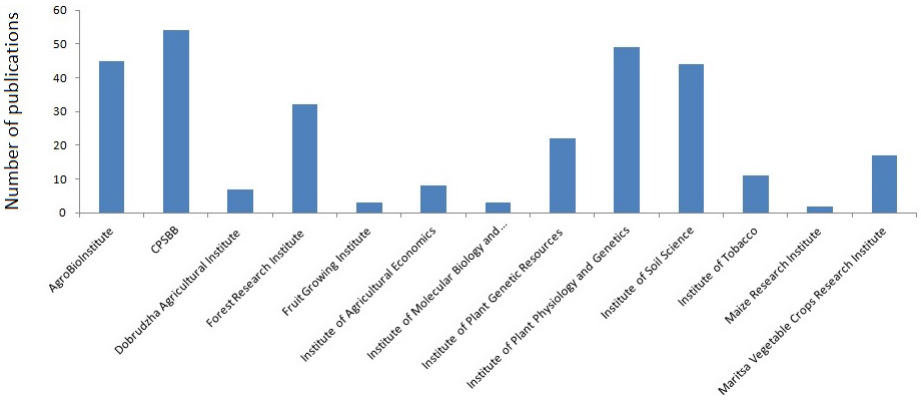
Peer reviewed research articles of Bulgarian plant science institutes published in impact factor journals in 2022. Source: SCOPUS database.

Fundamental and applied research is the main objective of CPSBB. At the very beginning, the CPSBB GA made the decision to adhere to the highest international standards and publish research articles only in medium and high impact factor journals. The same high standards were also applied when preparing collaborative national and international research projects. As a result of this, CPSBB displayed sustained growth over the years, as evidenced by the ever increasing number of publications and research projects ( Table 2). This growth was supported by the development of the modern infrastructure and human capacity. Starting with just four articles in its first years (2016–2017, Table 2), CPSBB substantially increased its publications to become the most productive plant science institute in 2022 ( Figure 2).

**Table 2.** Number of articles of the the Center of Plant Systems Biology and Biotechnology (CPSBB) in peer reviewed journals and projects in 2016 – 2023.

**Year**	**2016**	**2017**	**2028**	**2019**	**2020**	**2021**	**2022**	**2023**
**No. of publications**	4	4	22	23	72	76	54	43 *
**No. of projects**	1	1	2	3	4	5	8	19

* For the first half of 2023, 01.01.2023 – 30.06.2023

Not only the number of publications but also their quality distinguishes CPSBB. The vast majority of scientific articles are published in journals with medium and high impact factor. Example of high impact factor publications include articles in Nature Plants, Nature Genetics, Nature Communications, Nature Reviews Cell Biology, Plant Cell, PNAS USA, New Phytologist, etc.

Compared with the other Bulgarian research institutes from all fields, CPSBB is also among the best. Table 3 presents 46 Bulgarian research institutes, most of them part of either the Bulgarian Academy of Sciences (BAS) or the Bulgarian Agricultural Academy (BAA). The vast majority of these institutes are larger than CPSBB in term of scientific personnel and were established much earlier. For example, the Institute for Nuclear Research and Nuclear Energy and the Institute of Mathematics and Informatics have personnel of more than 300 people each, whereas CPSBB currently has personnel of 40 people. Nevertheless, CPSBB is in the top 10 in terms of number of peer reviewed articles in scientific journals, ranked number four by citations, and ranked number one by h-index for 2022 ( Table 3). According to the number of publications per personnel, CPSBB is number one in Bulgaria. All this demonstrates the quality of the scientific production and international visibility of CPSBB.

**Table 3.** Scientific output as measured by the number of peer reviewed research articles in 2022, citations of these publications, and the 2022 h-index of the Bulgarian research institutes. Data source: SCOPUS database.

Institutes	Publications	Citations	h-index
AgroBioInstitute	45	43	5
Center of Plant Systems Biology and Biotechnology	54	120	9
Central Laboratory of Applied Physics	12	4	1
Central Laboratory of Optical Storage and Processing of Information	1	0	0
Central Laboratory of Solar Energy and New Energy Sources	1	1	1
Centre of Biomedical Engineering	1	0	0
Dobrudzha Agricultural Institute General Toshevo	7	0	1
Forest Research Institute	32	24	3
Geological institute	47	24	3
Institute for Nuclear Research and Nuclear Energy	247	451	11
Institute of Agricultural Economics	8	1	1
Institute of Animal Science	15	2	1
Institute of Astronomy	59	34	4
Institute of Biodiversity and Ecosystem Research	210	151	8
Institute of Biophysics and Biomedical Engineering	152	107	7
Institute of Catalysis	35	15	4
Institute of Chemical Engineering	40	30	5
Institute of Electronics	118	88	5
Institute of Experimental Morphology and Anthropology	41	34	5
Institute of Fisheries and Aquaculture	2	0	1
Institute of General and Inorganic Chemistry	116	60	5
Institute of Information and Communication Technologies	248	91	6
Institute of Information Technologies	0	0	0
Institute of Mathematics and Informatics	272	247	8
Institute of Mechanics	92	46	6
Institute of Metal Science, Equipment and Technologies	35	5	2
Institute of Microbiology	102	72	7
Institute of Mineralogy and Crystallography	39	13	4
Institute of Molecular Biology	41	23	5
Institute of Molecular Biology and Biotechnology	3	9	2
Institute of Oceanology	22	14	3
Institute of Organic Chemistry with Centre of Phytochemistry	123	80	6
Institute of Plant Genetic Resources	22	4	1
Institute of Plant Physiology and Genetics	49	36	5
Institute of Physical Chemistry	53	23	4
Institute of Polymers	33	25	4
Institute of Sociology	0	0	0
Institute of Soil Science Agrotechnology and Plant Protection	44	13	2
Institute of Solid State Physics	100	51	5
Institute of Tobacco and Tobacco Products	11	4	2
Maize Research Institute	1	0	0
Maritsa Vegetable Crops Research Institute	17	10	2
National Institute of Geophysics Geodesy and Geography	49	25	3
National Institute of Meteorology and Hydrology	33	12	3
National Museum of Natural History	46	24	4
Space Research and Technology Institute	44	21	3

We also compared CPSBB with two leading European plant research centers: Max Plant Institute of Molecular Plant Physiology, Potsdam-Golm, Germany (MPIMP) and the John Innes Center, UK (JIC) ( Table 4). The data compared were number of publications, number of personnel, and number of publications per personnel. Both MPIMP and JIC are larger than CPSBB in terms of staff, but the number of scientific articles of CPSBB per person is two-fold higher than MPIMP and three-fold higher than JIC ( Table 4).

**Table 4.** Comparison of the Center of Plant Systems Biology and Biotechnology (CPSBB) with other Centers of Excellence in Europe. MPIMP, Max Planck Institute of Molecular Plant Physiology, Potsdam-Golm, Germany; JIC, John Innes Center, UK. The data about the number of publications (year 2022) and number of personnel is publicly available on the institutes’ web sites ( CPSBB; MPIMP; JIC).

Institute	Research publications	Personnel	Publications per person
CPSBB	54	40	1.35
MPIMP	194	298	0.65
JIC	175	427	0.41

### Funding

**Figure 3.**
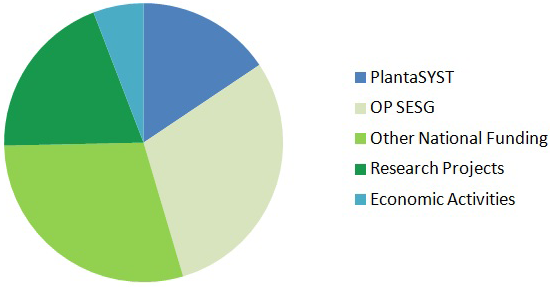
Funding sources of the Center of Plant Systems Biology and Biotechnology (CPSBB) according to the 10-year business plan (2017 – 2023). Incomes (51,300,000 EUR in total) are expected from the PlantaSYST project, from the Operational Programme “Science and Education for Smart Growth” (OP SESG), from other national funding (National Roadmap of research Infrastructure, Operational Programme “Programme Education”, third party funding from other research projects (Bulgarian National Science Fund, Horizon Europe, etc.), and economic activities coming from contracts with companies or services in the area of bioinformatics and plant metabolomics.

CPSBB has diversified its funding sources in order to reduce financial risks and secure long-term sustainability. There are incomes from the Bulgarian Government, the Framework Programmes of European Union (Horizon 2020, Horizon Europe), other international funding bodies, companies, services, and other economic and non-economic activities ( Figure 3). According to the 10-years business plan of CPSBB, the total funding for the period 2017 – 2027 is 51,000,000 EUR ( Figure 3).

***Funding from national sources.*** Thanks to the efforts of the CPSBB management, local authorities, and governmental officials, CPSBB was included in the National Roadmap of Research Infrastructure (NRRI) 2017 – 2027. This strategic document not only identifies the Bulgarian research institutes with utmost importance for the nation but more importantly provides the frame for long-term national funding. Through the NRRI, CPSBB received an initial funding of 300,000 BGN to purchase equipment in 2018. This seed money, together with the building provided by MVCRI, was vital for the first years of CPSBB.

With the establishment of the Operational Programme “Science and Education for Smart Growth” (OP SESG), CPSBB was invited to apply for national PlantaSYST co-funding. An investment project for the new research complex and for state of art equipment was prepared and submitted to the OP SESG and a co-funding contract for 30,000,000 BGN was signed in December 2019. The OP SESG co-funding project ends in December 2023. From 2024, a new six-year project of a similar size is expected from the new OP “Programme Education”. 

***Third party funding.*** Since its establishment, CPSBB has been very successful at attracting funds from international and national grants. According to the EU Open Data Portal 2019, CPSBB is the second largest Horizon 2020 beneficiary in Bulgaria out of 204 Bulgarian beneficiaries, behind only Sofia University. In Horizon Europe, the current EU framework programme, CPSBB has seven projects so far ( Table 1), which makes it again one of the most successful Bulgarian research institutes in the current framework programme. Additionally, CPSBB has four grants from the Bulgarian Nationas Science Fund (BNSF) and a number of other collaborative projects, amounting to 19 projects in total, amounting to more than 8,000,000 EUR in total ( Figure 3), which altogether contributes to the increased visibility and the sustainability of CPSBB.

***Collaboration with companies, services, and other economic activities.*** A number of economic activities coming from collaboration with biotechnology and plant breeding companies, as well as services in bioinformatics and metabolomics, are included in the 10-year business plan of CPSBB ( Figure 3). Over the years, CPSBB has established successful cooperation with a number of companies, including Aphaea.Bio (Belgium), BetterSeeds (Israel), BioAtlantis (Ireland), BGI (China), Huvepharma (Bulgaria), Ondo Solutions (Bulgaria), and Opora Zaden (Bulgaria). There are joint EU projects with some of these companies and/or joint research. Furthermore, framework partnership agreements and/or contracts were signed with more companies, e.g. GeoSemSelect (Bulgaria) and SUBA Seeds (Italy). It is expected that both the number of companies and their relative share in the incomes of CPSBB will increase in the near future, as the applied science develops and the number of technological solutions by CPSBB increase steadily.

The two service departments of CPSBB, Dept. of Bioinformatics and Dept. of Plant Metabolomics, are offering services not only to the other CPSBB research departments but also to external partner organizations, which adds to the economic activities of CPSBB ( Figure 3) and contributes to the long-term sustainability of the center. A number of research organizations and companies have requested such services and contracts for such services have already been signed with Plovdiv University and the Medical University of Plovdiv.

## Future outlook

In the future, CPSBB will continue to fulfill its important roles in the Bulgarian ecosystem. Most notably, it will strive to perform top level fundamental and applied research, establishing itself as a leading plant science center in Bulgaria. Secondly, it will play an even more prominent role in educating students and young researchers. More PhD students will enroll and graduate at CPSBB; joint MSc programs with the Agricultural University of Plovdiv are envisaged; teaching of BSc and MSc students from Plovdiv University in areas such as Plant Physiology will take place. Erasmus students are regularly visiting CPSBB. Furthermore, CPSBB is organizing various courses, workshops, and conferences open to PhD students and young postdocs. Finally, CPSBB will initiate formation of a plant science cluster with major universities, research organizations, and companies in Plovdiv, which will serve as a link between academia, industry, and farmer associations in the region.

## Conclusion

CPSBB has changed the research landscape in Bulgaria. Whereas most Bulgarian institutes are under the umbrella of the Bulgarian Academy of Sciences or the Bulgarian Agricultural Academy, CPSBB is a new type of fully autonomous institute with more flexibility and fast decision making. At the same time, CPSBB complies with all national and international high research and education standards, delivers PhD degrees in Biotechnology, cooperates with all major Bulgarian universities as well as many international partners, and fulfils its important role as a plant science hub in Plovdiv. Overall, CPSBB increased the scientific image of Bulgaria and contributed to the research and economic growth of the Plovdiv region. The keys to success lie in the full autonomy and high operational flexibility, the excellent infrastructure with state-of-the-art equipment, competitive salaries which allow selecting the best research, administrative, and technical personnel; the high internationalization, and the close cooperation with leading academic organizations, companies, and associations. Altogether, the development of CPSBB demonstrates that such types of institutes have a bright future in Bulgaria.

## Data Availability

All data underlying the results are available from the following websites: https://www.scopus.com/ https://cpsbb.eu/ https://www.mpimp-golm.mpg.de/2168/en https://www.jic.ac.uk/
