# Supplementary material for: Establishment and development of the Center of Plant Systems Biology and Biotechnology in Plovdiv, Bulgaria
Source: Open Res Eur. 2024 May 29;3:140. Originally published 2023 Sep 7. [Version 3] doi: 10.12688/openreseurope.16514.3 (PMC11153986; doi:10.12688/openreseurope.16514.3)
Supplement: Peer review history

# Reviewer report: Reviewer response for version 3

**DOI:** 10.21956/openreseurope.19189.r41042

**Published:** 2024-06-05

**Authors:** Panagiotis Moschou

**Affiliations:** 1 Department of Biology, University of Crete & Institute of Molecular Biology and Biotechnology, Foundation for Research and Technology, Heraklion, Greece

Authors considered all my comments. thanks!

Is the case presented with sufficient detail to be useful for teaching or other practitioners?

Yes

Is the work clearly and accurately presented and does it cite the current literature?

Partly

If applicable, is the statistical analysis and its interpretation appropriate?

Not applicable

Are all the source data underlying the results available to ensure full reproducibility?

Not applicable

Are the conclusions drawn adequately supported by the results?

Yes

Is the background of the case’s history and progression described in sufficient detail?

Yes

Reviewer Expertise:

plant biology, cell biology

I confirm that I have read this submission and believe that I have an appropriate level of expertise to confirm that it is of an acceptable scientific standard.

# Reviewer report: Reviewer response for version 2

**DOI:** 10.21956/openreseurope.18559.r37814

**Published:** 2024-04-22

**Authors:** Jose M. Pardo

**Affiliations:** 1 Institute of Plant Biochemistry and Photosynthesis, Consejo Superior de Investigaciones Cientificas and Universidad de Sevilla, Sevilla, Spain

The paper has been updated as requested.

 No further requests or comments.

Is the case presented with sufficient detail to be useful for teaching or other practitioners?

Yes

Is the work clearly and accurately presented and does it cite the current literature?

Yes

If applicable, is the statistical analysis and its interpretation appropriate?

Not applicable

Are all the source data underlying the results available to ensure full reproducibility?

Not applicable

Are the conclusions drawn adequately supported by the results?

Yes

Is the background of the case’s history and progression described in sufficient detail?

Yes

Reviewer Expertise:

Plant stress physiology

I confirm that I have read this submission and believe that I have an appropriate level of expertise to confirm that it is of an acceptable scientific standard.

# Reviewer report: Reviewer response for version 2

**DOI:** 10.21956/openreseurope.18559.r38656

**Published:** 2024-03-13

**Authors:** Panagiotis Moschou

**Affiliations:** 1 Department of Biology, University of Crete & Institute of Molecular Biology and Biotechnology, Foundation for Research and Technology, Heraklion, Greece

1."Bulgaria is one of the Widening countries, together with Portugal, Luxemburg, the countries that joined the European Union (EU) after 2004, and several Horizon 2020 associated states": the authors should state for a broader audience, which are these countries and the recent additions (e.g., Greece).

 2. What part of Bulgarian GDP is dedicated to research? 

 3. Are there specific strategic points for development? Authors state vaguely, "there was a need for a new research institute to study the rapidly evolving field of plant systems biology and to apply plant biotechnology approaches to solve important challenges such as food production or production of plant-derived metabolites with medical and pharmaceutical applications."

 4. Authors do not explain what "teaming" is for the general audience

 5. Table 1: would benefit if authors include the budget in euros.

 6. I would prefer to see details on this equipment: state of the art metabolomics and biotechnology machines (ICP-MS, GC-MS, UHPLC-MS, pilot-scale bioreactor, confocal laser microscope, state of art plant growth chambers, etc.).

 7. What is the ranking of Bulgaria in research outputs? Furthermore, there are Bulgarian journals of international standing that involve plant research and they could be upgraded form such actions. A mention on that is worthwhile. 

 8. "publish research articles only in medium and high impact factor journals. ". What are some examples? "The vast majority of scientific articles are published in journals with medium and high impact factor. Example of high impact factor publications include articles in Nature Plants, Nature Genetics, Nature Communications, Nature Reviews Cell Biology, Plant Cell, PNAS USA, New Phytologist, etc." This should move up a bit.

 9. Figure 2: should be normalized per something (personnel, budget, size etc.)

 10. I do not think it makes sense to compare to institutes that are of completely different disciplines. Furthermore, how many institutes are there in total in Bulgaria?

 11. Table 4 is only quantitative. I would prefer qualitative data, as well. These are, of course, top-notch institutes and the comparison is a bit unfair. Why not pick other EU institutes that are within reach?

 12. 300,000 and 30,000,000 BGN: in euros?

 13. Is there income from " ***Collaboration with companies, services, and other economic activities."***, at the moment? 

 14. Figure 3: missing % values on the pie chart.

Is the case presented with sufficient detail to be useful for teaching or other practitioners?

Yes

Is the work clearly and accurately presented and does it cite the current literature?

Partly

If applicable, is the statistical analysis and its interpretation appropriate?

Not applicable

Are all the source data underlying the results available to ensure full reproducibility?

Not applicable

Are the conclusions drawn adequately supported by the results?

Yes

Is the background of the case’s history and progression described in sufficient detail?

Yes

Reviewer Expertise:

Plant Biology, Plant Cell Biology

I confirm that I have read this submission and believe that I have an appropriate level of expertise to confirm that it is of an acceptable scientific standard, however I have significant reservations, as outlined above.

# Response

**Published:** 2024-05-07

**Authors:** Tsanko Gechev

**Affiliations:** Center of Plant Systems Biology and Biotechnology, Plovdiv, Plovdiv Province, Bulgaria

We thank the reviewer for his valuable comments and suggestions. We have revised the article, addressing all reviewers’ points. The new additions/revised text is marked in red color.   1."Bulgaria is one of the Widening countries, together with Portugal, Luxemburg, the countries that joined the European Union (EU) after 2004, and several Horizon 2020 associated states": the authors should state for a broader audience, which are these countries and the recent additions (e.g., Greece). Response: The list of Widening countries was added, including Greece as a new addition in Horizon Europe.   2. What part of Bulgarian GDP is dedicated to research?

 Response: The Bulgarian GDP dedicated to research is 0.77 – this is now mentioned in the article and compared to the EU average of 2.4.

 3. Are there specific strategic points for development? Authors state vaguely, "there was a need for a new research institute to study the rapidly evolving field of plant systems biology and to apply plant biotechnology approaches to solve important challenges such as food production or production of plant-derived metabolites with medical and pharmaceutical applications."

 Response: Some of the strategic points for scientific development of Bulgaria are listed now in the article, namely Biotechnology and Human Health.

 4. Authors do not explain what "teaming" is for the general audience

 Response: Teaming was explained for the general audience.

 5. Table 1: would benefit if authors include the budget in euros.

 Response: We have included the budget in euros.

 6. I would prefer to see details on this equipment: state of the art metabolomics and biotechnology machines (ICP-MS, GC-MS, UHPLC-MS, pilot-scale bioreactor, confocal laser microscope, state of art plant growth chambers, etc.).

 Response: We have provided more information on some o the state of the art equipment, including technical details and the manufacturers/country of origin.

 7. What is the ranking of Bulgaria in research outputs? Furthermore, there are Bulgarian journals of international standing that involve plant research and they could be upgraded form such actions. A mention on that is worthwhile.

 Response: The Bulgaria’s research output is below 70% of the EU average, hence it is a Widening country. There are Bulgarian journals ranked internationally; however, their IF is low – CPSBB does not publish in journals with low IF.

 8. "publish research articles only in medium and high impact factor journals. ". What are some examples? "The vast majority of scientific articles are published in journals with medium and high impact factor. Example of high impact factor publications include articles in Nature Plants, Nature Genetics, Nature Communications, Nature Reviews Cell Biology, Plant Cell, PNAS USA, New Phytologist, etc." This should move up a bit.

 Response: The sentence “The vast majority of scientific articles are published in journals with medium and high impact factor.” Was moved up, as recommended.

 9. Figure 2: should be normalized per something (personnel, budget, size etc.)

 Response: The publications in figure 2 are normalized per personnel and the data updated for 2023.

 10. I do not think it makes sense to compare to institutes that are of completely different disciplines. Furthermore, how many institutes are there in total in Bulgaria?

 Response: We agree with the reviewer that there is no sense to compare institutes that are of completely different disciplines. Hence, we compared only plant science institutes. There are 90 institutes in total in Bulgaria: 48 of them are part of the Bulgarian Academy of Sciences, 25 are part of the Bulgarian Agricultural Academy, and the rest are independent institutes funded either by the Government or private research institutes.

 11. Table 4 is only quantitative. I would prefer qualitative data, as well. These are, of course, top-notch institutes and the comparison is a bit unfair. Why not pick other EU institutes that are within reach?

 Response: We have intently selected these institutes, as they are indeed top institutes and publish, as CPSBB, high level research in medium and high ranked journals. The rankings of the journals in which the selected international institutes publish their research are fully comparable/the same as CPSBB. We do not want to compare CPSBB with international institutes which publish scientific articles in low IF journals.

 12. 300,000 and 30,000,000 BGN: in euros? The numbers are given in both BGN and in euros, as well.

 13. Is there income from " ***Collaboration with companies, services, and other economic activities."***, at the moment? 

 Response: Yes, there is such an income. It is included in Figure 3 as “Economic activities” and is better clarified in the text now.  

 14. Figure 3: missing % values on the pie chart.

 Response: The percentage values are now given in the legend of the pie chart.

# Reviewer report: Reviewer response for version 1

**DOI:** 10.21956/openreseurope.17828.r36523

**Published:** 2023-12-28

**Authors:** Jose M. Pardo

**Affiliations:** 1 Institute of Plant Biochemistry and Photosynthesis, Consejo Superior de Investigaciones Cientificas and Universidad de Sevilla, Sevilla, Spain

This article presents a detailed account of the establishment, organizational structure, funding, research interests and the placement within the European research landscape of the Center of Plant Systems Biology and Biotechnology (CPSBB) in Plovdiv, Bulgaria. The data and facts presented are both clear and comprehensive. The main message is that through the join venture of national and local administrative branches and with the financial support of the Horizon 2020 framework program, the CPSBB is well positioned to become a leading institution of the areas of biotechnology and plant systems biology in Bulgaria.

 Just a few remarks to improve the text:

 1. When describing the different Departments of CPSBB, the Department name should have an embedded link to the corresponding web page.

 2. Table 1. Mispelled 'Coordinator' in several rows.

 3. Sentence 'Currently, according to the percentage of foreign researchers, CPSBB is the most international Bulgarian research institute' should include the actual percent in CPSBB.

 4. Please, for the general readership, translate every figure in BGN currency to EUR.

Is the case presented with sufficient detail to be useful for teaching or other practitioners?

Yes

Is the work clearly and accurately presented and does it cite the current literature?

Yes

If applicable, is the statistical analysis and its interpretation appropriate?

Not applicable

Are all the source data underlying the results available to ensure full reproducibility?

Not applicable

Are the conclusions drawn adequately supported by the results?

Yes

Is the background of the case’s history and progression described in sufficient detail?

Yes

Reviewer Expertise:

Plant stress physiology

I confirm that I have read this submission and believe that I have an appropriate level of expertise to confirm that it is of an acceptable scientific standard.

# Response

**Published:** 2024-01-21

**Authors:** Tsanko Gechev

**Affiliations:** Center of Plant Systems Biology and Biotechnology, Plovdiv, Plovdiv Province, Bulgaria

The authors thank to the reviewer for his valuables remarks, comments, and corrections.   Reviewer’s remarks: 

 1. When describing the different Departments of CPSBB, the Department name should have an embedded link to the corresponding web page.

   Answer: Embedded link to the Departments’ names were included in the article.   

 2. Table 1. Mispelled 'Coordinator' in several rows. Corrected 

 3. Sentence 'Currently, according to the percentage of foreign researchers, CPSBB is the most international Bulgarian research institute' should include the actual percent in CPSBB. The current actual percentage was included. 

 4. Please, for the general readership, translate every figure in BGN currency to EUR. The value in EUR was added in brackets next to the value in BGN.
